# Transferability of genetic risk scores in African populations

**DOI:** 10.1038/s41591-022-01835-x

**Published:** 2022-06-02

**Authors:** Abram B. Kamiza, Sounkou M. Toure, Marijana Vujkovic, Tafadzwa Machipisa, Opeyemi S. Soremekun, Christopher Kintu, Manuel Corpas, Fraser Pirie, Elizabeth Young, Dipender Gill, Manjinder S. Sandhu, Pontiano Kaleebu, Moffat Nyirenda, Ayesha A. Motala, Tinashe Chikowore, Segun Fatumo

**Affiliations:** 1 The African Computational Genomics (TACG) Research Group, MRC/UVRI and LSHTM, Entebbe, Uganda; 2 Malawi Epidemiology and Intervention Research Unit, Lilongwe, Karonga Malawi; 3 Sydney Brenner Institute for Molecular Bioscience, Faculty of Health Sciences, University of the Witwatersrand, Johannesburg, South Africa; 4 African Centre of Excellence in Bioinformatics, University of Science and Technologies of Bamako, Bamako, Mali; 5 Department of Pathology and Molecular Medicine, McMaster University, Michael G. DeGroote School of Medicine, Hamilton, Ontario Canada; 6 Corporal Michael J. Crescenz VA Medical Center, Philadelphia, PA USA; 7 Hatter Institute for Cardiovascular Diseases Research in Africa (HICRA), Department of Medicine, University of Cape Town, Cape Town, South Africa; 8 Population Health Research Institute, David Braley Cardiac, Vascular and Stroke Research Institute, Hamilton, Ontario Canada; 9 Cambridge Precision Medicine Limited, ideaSpace, University of Cambridge Biomedical Innovation Hub, Cambridge, United Kingdom; 10 Institute of Continuing Education, Madingley Hall, University of Cambridge, Cambridge, UK; 11 Facultad de Ciencias de la Salud, Universidade Internacional de La Rioja, Madrid, Spain; 12 Department of Diabetes and Endocrinology, University of KwaZulu-Natal, Durban, South Africa; 13 Omnigen Biodata Ltd, Cambridge, UK; 14 Department of Epidemiology and Biostatistics, School of Public Health, Imperial College London, London, United Kingdom; 15 Clinical Pharmacology and Therapeutics Section, Institute of Medical and Biomedical Education and Institute for Infection and Immunity, St George’s, University of London, London, UK; 16 MRC/UVRI and LSHTM, Entebbe, Uganda; 17 MRC/Wits Developmental Pathways for Health Research Unit, Department of Paediatrics, Faculty of Health Sciences, University of the Witwatersrand, Johannesburg, South Africa; 18 London School of Hygiene and Tropical Medicine, London, UK; 19 H3Africa Bioinformatics Network (H3ABioNet) Node, Centre for Genomics Research and Innovation, NABDA/FMST, Abuja, Nigeria

**Keywords:** Medical genetics, Genomics

## Abstract

The poor transferability of genetic risk scores (GRSs) derived from European ancestry data in diverse populations is a cause of concern. We set out to evaluate whether GRSs derived from data of African American individuals and multiancestry data perform better in sub-Saharan Africa (SSA) compared to European ancestry-derived scores. Using summary statistics from the Million Veteran Program (MVP), we showed that GRSs derived from data of African American individuals enhance polygenic prediction of lipid traits in SSA compared to European and multiancestry scores. However, our GRS prediction varied greatly within SSA between the South African Zulu (low-density lipoprotein cholesterol (LDL-C), *R*^2^ = 8.14%) and Ugandan cohorts (LDL-C, *R*^2^ = 0.026%). We postulate that differences in the genetic and environmental factors between these population groups might lead to the poor transferability of GRSs within SSA. More effort is required to optimize polygenic prediction in Africa.

## Abstract (web summary)

A new study reveals that polygenic scores for lipid traits derived from data of African American individuals have high predictive value in a South African Zulu cohort but are poor predictors in a cohort from Uganda, further highlighting the need to improve polygenic predictions in populations of African ancestries.

## Main

Genome-wide association studies (GWASs) have successfully identified and characterized genetic variants associated with lipid traits^1–3^. To date, roughly 700 single-nucleotide polymorphisms (SNPs) are associated with various lipid traits^3–9^. These discoveries are now beginning to unravel the biology of dyslipidemia and aid prediction for precision medicine. To date, polygenic risk across the genome can be aggregated by generating genome-wide weighted scores to predict the risk of a disease in an independent population^10,11^. However, most lipid trait discoveries have been made in European or Asian ancestries^4–9^. Genetic risk scores (GRSs) derived from European ancestry tend to perform poorly in genetically diverse populations, including Africans^10^, probably due to unique differences in linkage disequilibrium (LD) patterns, allele frequencies and environmental exposures^12^ between different populations. Lack of precise GRSs in Africans hinders risk stratification and targeted treatments essential for precision medicine and may exacerbate health disparities.

Recent studies have indicated that using multiancestry summary statistics enhance GRS performance in diverse populations^13^. Moreover, previous studies suggested that using summary statistics from African Americans may improve GRS performance in sub-Saharan Africans^14^. We, therefore, undertook a study to determine the best approach for lipid traits polygenic risk prediction, including low-density lipoprotein cholesterol (LDL-C), high-density lipoprotein cholesterol (HDL-C), triglycerides (TGs) and total cholesterol (TC) in sub-Saharan Africans using publicly available GWAS summary statistics. This study assessed the performance, portability and predictivity of GRSs derived from data of African Americans, Europeans and multiancestry (African American, European and Hispanic American) individuals in Ugandan and South African Zulu cohorts.

**Fig. 1:**
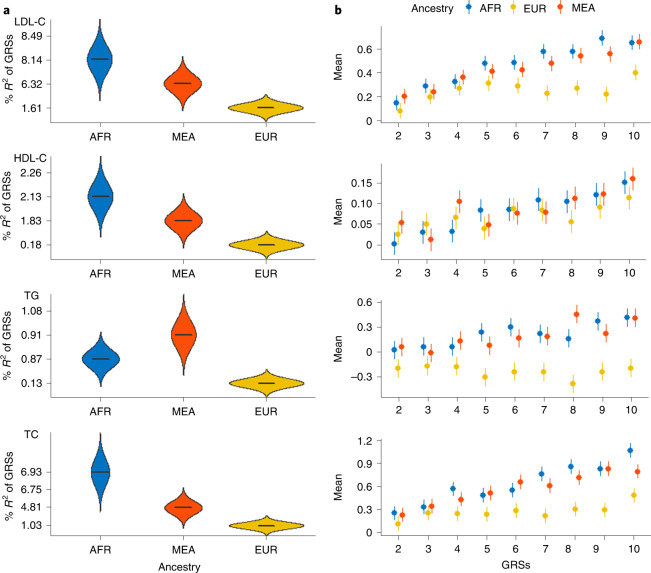
Performance of GRSs for lipid traits in the South African Zulu cohort using the MVP GWAS summary statistic results of various ancestry populations, including individuals of African American, European and multiethnic ancestry populations. **a**, Violin plots showing GRSs that explained the highest proportion of variance (*R*^2^) for lipids derived from African American (AFR), European (EUR) and multiancestry (MEA) populations. **b**, GRSs in deciles compared to the first decile. The *y* axis shows the mean, and the *x* axis is the GRSs in deciles. The points show mean, and error bars represent standard errors of the mean. All South African Zulu cohorts (*n* = 2,598) were used in this analysis.

**Extended Data Fig. 1:**
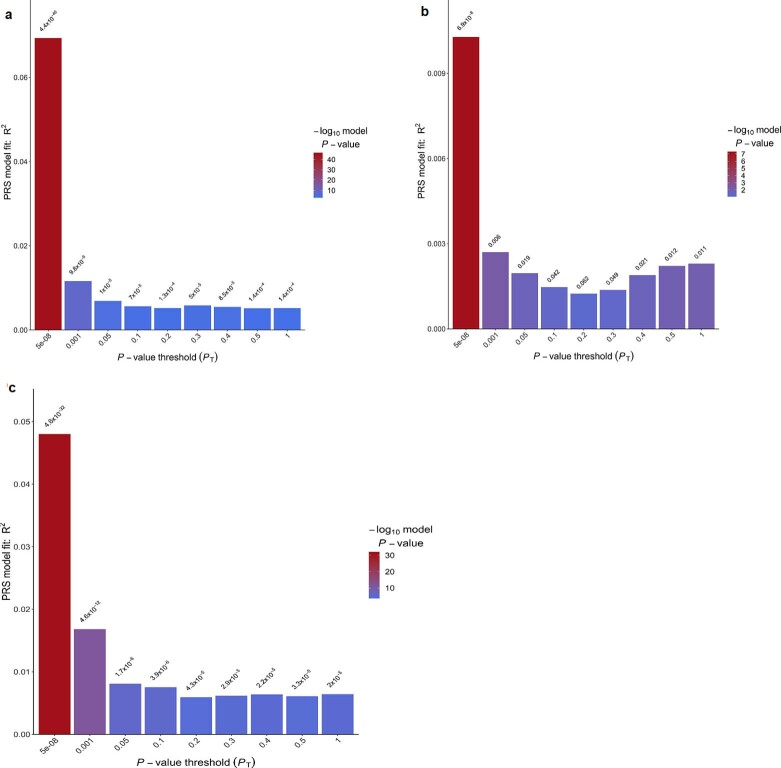
Proportion of variance of TC explained by GRS in the South African Zulu samples using GRS derived from GWAS. (a) African American ancestry, (a) European ancestry and (a) multiethnic ancestry. The bars represent GRS calculated for subsets of markers at different p-value thresholds. The best GRS in red color was selected based on having the highest proportion of the variance (R2) for the trait in linear models adjusted for age, sex and principal components.

**Extended Data Fig. 2:**
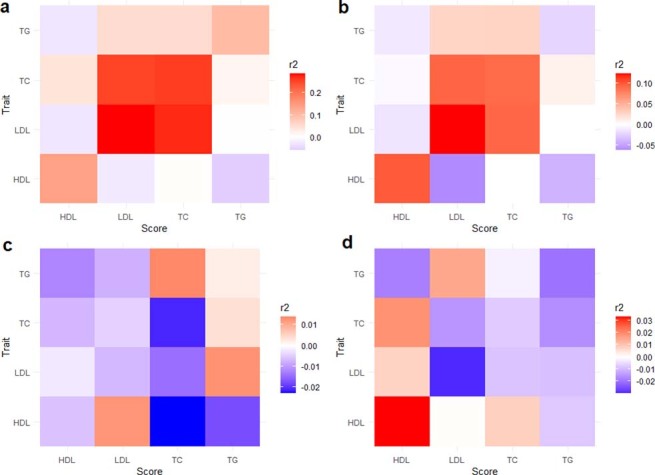
Correlation coefficients between GRS and serum lipid levels. (a) African American derived GRS in the South African Zulu dataset. (b) European derived GRS in the South African Zulu. (c) African American derived GRS in the Ugandan cohort. (d) European derived GRS in the Ugandan cohort. The correlation coefficients r2 are given with colors corresponding to the direction and strength of r2. The r2 on the diagonal represents the strength of correlation of a GRS with its target lipid trait. The off-diagonal r2 represents the strength of correlation of a GRS with other lipid traits.

We computed GRSs using PRSice-2. Of the many GRSs computed at various *P*-value thresholds that ranged from 1 to 5 × 10^−8^, the GRS that explained the highest proportion of variance (*R*^2^) in any trait for the African, European and multiancestry populations (Methods) was selected as the best-performing one (Extended Data Table 1 and Extended Data Fig. 1). In the South African Zulu cohort, the best-performing GRSs for LDL-C was African American (*R*^2^ = 8.14%, *P*-value threshold (*P*_T_) < 5 × 10^−8^), followed by the multiancestry approach (derived from individuals of African ancestry, European ancestry and Hispanic American) (*R*^2^ = 6.32%, *P*_T_ < 5 × 10^−08^), and the one from individuals of European ancestry (*R*^2^ = 1.61%, *P*_T_ < 5 × 10^−08^, Fig. 1a and Extended Data Table 2). Although the African American-derived GRS predicted better in the South African Zulu cohort its prediction was lower in Ugandan cohort (Extended Data Table 3). Moreover, our African American-derived GRSs (coefficient ranging from 0.100 to 0.286) were better correlated with all serum lipid levels than the European GRSs (coefficients ranging from 0.091 to 0.123) in South African Zulu (Extended Data Fig. 2).

We proceeded to evaluate risk stratification based on the deciles of the GRSs for the lipid traits presented (Methods). We compared the effect sizes of serum lipid levels from the first GRS decile after correction for age, sex and ten principal components. In parallel, we observed that individuals in the top 10% of the GRSs had higher serum lipid levels than those in the first decile (Fig. 1b). Notably, multiancestry-derived GRS was the best-performing approach for HDL-C and TG (Fig. 1b). Individuals at the top 10% of the GRSs had a higher difference of 0.16 mmol liter^−1^ and 0.45 mmol liter^−1^ for HDL-C and TG levels, respectively, compared to individuals at the bottom 10% GRSs. For LDL-C and TC, the best-performing approach was the African American GRS, with a mean difference (first versus tenth decile) of 0.70 mmol liter^−1^ for LDL-C and 1.09 mmol liter^−1^ for TC (Fig. 1b) for those at the top 10% GRS decile.

**Fig. 2:**
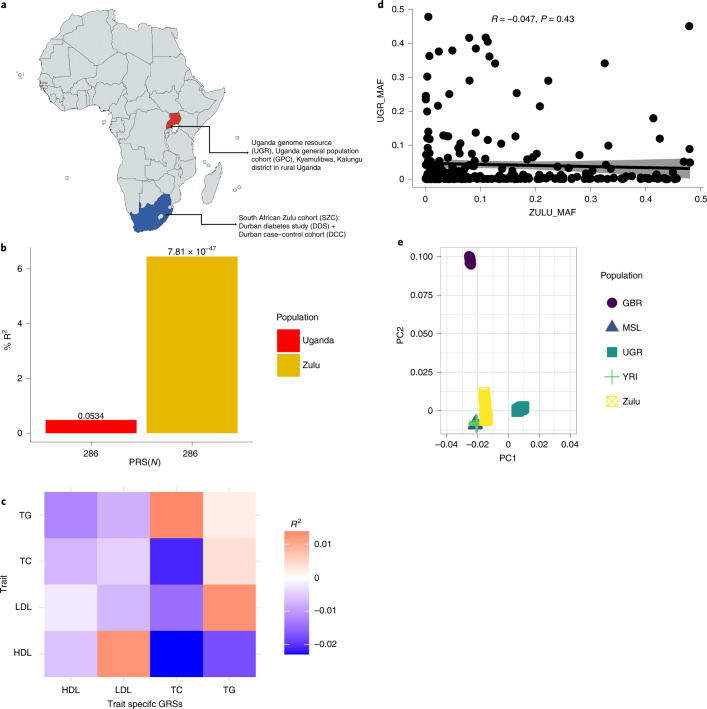
GRSs of individuals of African ancestry with dyslipidemia. **a**, Map of Africa showing sample collection points in Kyamulibwa in Kalungu district, Uganda and Durban, Kwazulu-natal province, South Africa**. b**, Bar plot showing comparative performance of polygenic prediction of TC using the same GRS comprising 286 SNPs, which was developed in Ugandan cohort (*n* = 6,407) and then replicated in the South African Zulu cohort (*n* = 2,598). The *y* axis is the prediction accuracy (*R*^2^), and the *x* axis is the number of SNPs in the GRS for TC used. **c**, Correlation coefficients between African American-derived GRSs and serum lipid levels in the Ugandan cohort. **d**, Scatter plot for the correlation of the same minor allele frequencies (MAF) between the South African Zulu and Ugandan cohorts (*R* = Pearson correlation, one-sided test). PC, principal component. **e**, Scatter plot for the principal component analysis of the 1000 Genomes Project reference populations with the South African Zulu and Ugandan cohorts (GBR, British; MSL, Mende; UGR, Uganda genome resource; Zulu, South African Zulu; YRI, Yoruba).

We proceeded to evaluate the transferability of a GRS derived from an African American cohort in Ugandan and South African Zulu cohorts (Fig. 2a). Using TC as an example, we noted that the same African American GRS of 286 SNPs performed poorly in the Ugandan cohort (*R*^*2*^ = 0.045%) but much better in the South African Zulu cohort (6.345%) (Fig. 2b). The correlations of the GRS with lipid traits were lower among the Ugandan cohort compared to the South African Zulu cohort (Fig. 2c). Of all the lipid traits, predictability was lowest for TGs, possibly due to the nonfasting of participants before blood collection for lipid analysis. TGs, unlike TC and HDL, are sensitive to dietary intake, which might have affected their accurate estimation and consequently its prediction^15^.

**Extended Data Fig. 3:**
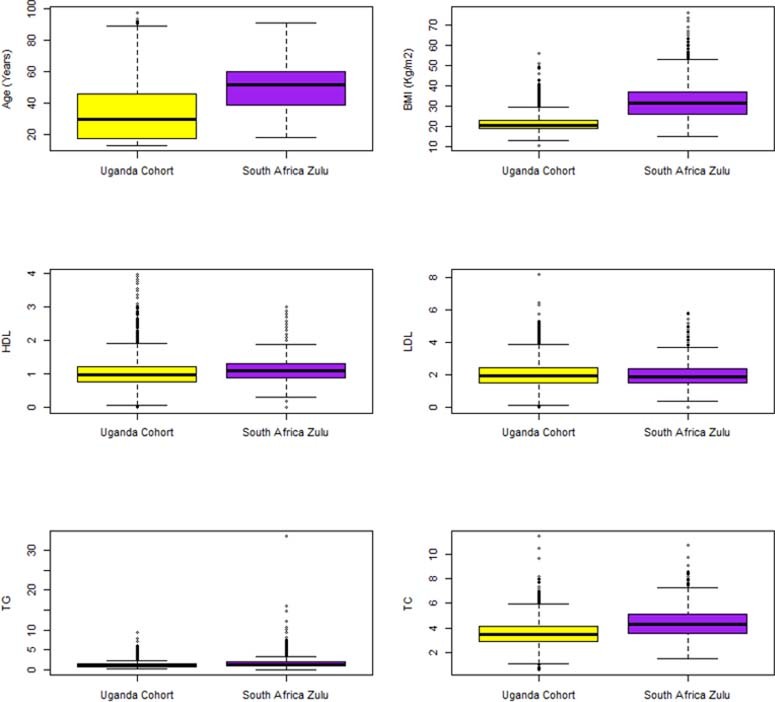
Box plots showing the distribution of age, BMI and lipid traits among the Ugandan and South African Zulu cohorts. The horizontal line is the median values, error bars are 25th and 75th percentiles. Extreme values are maximum and minimum for respective traits. Data analysis were performed in all Ugandan (n = 6,407) and South African Zulu (n = 2,598) cohorts.

We then sought to evaluate the contribution of minor allele frequencies to the poor transferability of the GRSs between the Ugandan and South African Zulu cohorts. We compared allele frequencies of the SNPs in the African American-derived GRSs in the Ugandan and South African Zulu cohorts (Fig. 2d). We noted that there were marked differences in age, body mass index and allele frequencies between these cohorts, which might have contributed to the poor transferability of the African American GRS (Extended Data Fig. 3 and Fig. 2e). The South African Zulu cohort recruited participants from an urbanized setting compared to the Ugandan cohort. Therefore the urban and rural environmental differences might also be playing a part in the poor transferability of the African American-derived GRSs between the Ugandan and South African Zulu cohorts. This finding suggests that both genetic and environmental factors might be responsible for the differences in the performance of GRSs in the Ugandan cohort.

**Extended Data Fig. 4:**
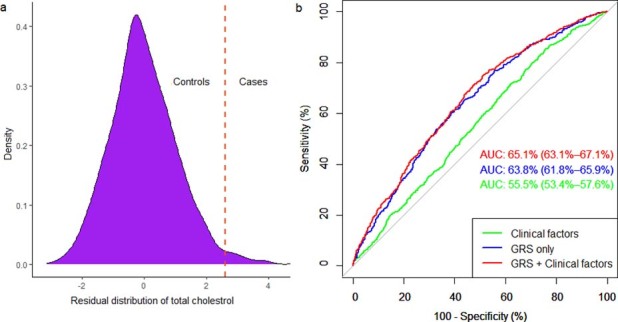
The discriminative power of our polygenic risk score or GRS to successfully identify the individuals of African ancestry with dyslipidaemia. (a) Distribution of total cholesterol (TC) among South African Zulus. The top 10% deciles were named “cases,” and the lower deciles were designated as “controls.” (b) The area under the curve in South African Zulu.

Next, we then assessed the ability of the GRS to identify people with high lipid levels compared to conventional risk factors. We computed residuals of the linear model of TC adjusted for age and sex in the South African Zulu cohort. We then selected individuals at the top 10% of the residual density plot as ‘cases’ and the remaining 90% deciles as ‘controls’ (Extended Data Fig. 4a). For example, the average TC level in cases was 6.51 mmol liter^−1^ compared to 4.30 mmol liter^−1^ in controls, representing a difference of 2.21 mmol liter^−1^. Using logistic regression models, we evaluated the prediction of the African American GRSs trained from the Ugandan cohort in the South African Zulu cohort. The areas under the curve were 55.5% (95% confidence interval [CI], 53.4–57.6%) for clinical factors, including type 2 diabetes, body mass index, age, sex and five principal components, and 63.8% (95% CI, 61.8–65.9%) for GRSs only (Extended Data Fig. 4b). Moreover, the net reclassification index for the model of the clinical factors increased by 42% after adding the GRSs to this model, further supporting our results that the GRS was better at identifying individuals with high TC compared to conventional clinical factors. However, lipid profiles rather than conventional risk factors are used to assess for dyslipidemia in the clinical setting. Lipid profiles are easier to collect and interpret than GRSs, thereby limiting the clinical application of the GRS. Nonetheless, GRSs might find use in the risk stratification of children and young adults long before they start to exhibit elevated lipid levels^15^.

Consistent with previous reports, GRSs derived from individuals of African ancestry performed significantly better in sub-Saharan Africans than GRSs derived from individuals of European ancestry^10,16–18^. The performance of GRS derived from data of African American individuals for LDL-C (*R*^2^ = 8.14%) was much higher than the performances reported by Johnson et al. (ranging from 1.99% to 4.48% in African American, Asian American, white and Hispanic individuals for LDL-C)^18^. This difference suggests that GRSs computed using African Ancestry discovery GWASs may lead to better polygenic predictions of lipids in individuals of African descent. However, continental Africans are characterized by high genetic diversity, which may affect the performance and transferability of GRSs within Africa^12^.

Moreover, our results suggest poor transferability of GRS between South African Zulu and Ugandan populations. This might be due to differences in environmental (Extended Data Fig. 3) and genetic factors (Fig. 2D) between the South African Zulu and Ugandan cohorts^19,20^. The poor performance of GRS within the same ancestry population hinders the implementation of GRS in preventative healthcare. It may lead to inaccurate results when applied to different ethnic groups within sub-Saharan Africa. This further suggests the need for more efforts to optimize polygenic prediction in Africa. A limitation of this study is the none inclusion of diet and regular physical activity for the prediction of dyslipidemia. Nevertheless, we included crucial clinical factors, including body mass index, which is strongly associated with diet and regular physical activity; hence, the overall performance of our GRSs were robust.

In conclusion, using GRSs derived from data of individuals of African ancestry performed better in predicting lipid traits in sub-Saharan African populations than GRSs derived from data of individuals of European ancestry. However, the GRS are likely to have variable performances across sub-Saharan African populations, as shown by the differences seen between South African Zulu and Ugandan populations.

## Methods

### Study population

The target data for GRS construction were taken from the South African Zulu cohort, a combination of the Durban Diabetes Study (DDS) and the Durban Case-Control Study (DCC) KwaZulu-Natal South Africa. DDS is a population-based cross-sectional study of individuals aged >18 years residing in the urban black communities in Durban, KwaZulu-Natal, South Africa. DCC is a case–control study of individuals aged >40 years with diabetes recruited from tertiary hospitals in Durban. Data collection was conducted from 2009 to 2013 for the DCC and from 2013 to 2014 for the DDS. The survey questionnaire included socioeconomic factors, health information, lifestyle factors, blood pressure, anthropometric measurements (including height, weight, and hip and waist circumferences), biomarkers for communicable and noncommunicable diseases and genetic data. Of the 2,804 individuals surveyed, 1,204 were from the DDS and 1,600 were from the DCC; more detailed information on the study design and quality controls has been published previously^21,22^. Informed consent was obtained from all DDS and DCC participants. The DDS was approved by the University of KwaZulu-Natal Biomedical Research Ethics Committee (BF030/12) and the UK National Research Ethics Service (14/WM/); the DCC was approved by the University of KwaZulu-Natal Biomedical Research Ethics Committee (BF078/08) and the UK National Research Ethics Service (11/H0305/6).

The comparative cohort was taken from the Uganda genome resource (UGR), which is the genomic and phenotypic resource generated from the Uganda General Population Cohort (GPC). The GPC is a population-based cohort study founded in the late 1980s, and it has over 22,000 participants from 25 neighboring villages in Kyamilibwa in rural Uganda. This open-cohort study was established to investigate the trends of HIV infection in Uganda. However, the cohort’s focus now is to examine the role of host genetic variants associated with communicable and noncommunicable diseases in rural Ugandans^22^. Informed consent was obtained from all participants, and the Uganda GPC was approved by Uganda Virus Research Institute Research and Ethics Committee (UVRI-REC HS 1978) and the Uganda National Council for Science and Technology (UNCST SS 4283).

### Measurement of lipid traits

Nonfasting serum lipid levels were measured using the Cobas Integra 400 Plus Chemistry analyzer (Roche Diagnostics), an automated analyzer that uses four different technologies: absorption photometry, fluorescence polarization immunoassay, immune turbidimetry and potentiometry for accurate analysis. HDL-C and LDL-C were measured using the homogeneous enzymatic colorimetric assays^23,24^.

### Polygenic risk score

GWAS meta-analysis summary statistics results from the MVP were used as the discovery data sets in GRS computation for the specific lipids. For instance, LDL-C summary statistics from the multiancestry, African American and European cohorts were used for the development of the LDL-C GRSs. The MVP summary statistics results comprised an average of 30 million SNPs from more than 800,000 individuals of diverse ancestry. Of these, 61,796 were African American, and 241,54 were European. The multiancestry summary statistics comprised 25,747 individuals from Hispanic American, European and African American populations. Methods used for genotyping and quality control of MVP data have been previously described^25^.

For GRS construction, SNPs from MVP serum lipid summary statistics were clumped based on their LD. We clumped SNPs at different *R*^2^ thresholds, and a 500-kb clumping window with *R*^2^ of 0.5 proved to be the best-fitting and best-performing model for all lipid traits. We also tested the best P-value threshold for selecting which clumped SNPs we would include in the final GRS for the range of 1 to 5 × 10^−8^. The *P*-value threshold, which accounted for the highest proportion of the variance of the trait *R*^2^, was selected as the best GRS for TC. The GRS was calculated by multiplying the weight of the SNPs with the number of risk alleles (0/1/2) carried by each individual using the algorithm implemented in the PRSice-2 software^26^. The GRS generated was incorporated into the generalized linear regression model to explain the serum lipids’ performance while adjusting for age, sex, type 2 diabetes and five principal components, which were calculated using unrelated individuals and on pruned genotyped data sets using PLINK. An incremental *R*^2^ was computed from each model by the PRSice algorithm and plotted against the *P*_T_. *R*^2^ is the difference between the *R*^2^ of the fully adjusted model (GRS, age, sex, five principal components and diabetes status) and the *R*^2^ of the null model (age, sex, five principal components and diabetes status); the best GRS achieved the highest proportion of *R*^2^ (Fig. 1a).

The best-performing GRS was then categorized into deciles. The bottom decile was used as a reference and compared to other deciles. The difference in the effect sizes of the lipid levels across different GRS deciles was tested using linear regression while adjusting for age, sex, five principal components and diabetes status. We then performed logistic regression with the top decile of the GRS as cases with the remaining 90% as controls. The output of the logistic regression was used to compute the receiver operating curves in R. Furthermore, we used a net reclassification index to assess the ability of the GRS to identify individuals with high TC in the South African Zulu cohort. This reclassification was done by comparing the improvement in reclassification of a null model that comprised the conventional risk factors with that of a null model plus the GRSs using the PredictAbel package in R. The performance of the GRS from each lipid trait was compared among individuals of African ancestry, European ancestry and multiethnic ancestry populations using the ggplot2 R statistical package^27,28^.

### Reporting summary

Further information on research design is available in the Nature Research Reporting Summary linked to this article.

## Online content

Any methods, additional references, Nature Research reporting summaries, source data, extended data, supplementary information, acknowledgements, peer review information; details of author contributions and competing interests; and statements of data and code availability are available at 10.1038/s41591-022-01835-x.

### Supplementary information

Reporting Summary

## Figures and Tables

**Extended Data Table 1:**
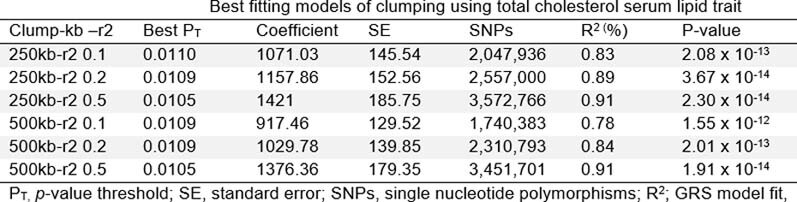
Best-fitting models of clumping using the TC serum lipid trait

**Extended Data Table 2:**
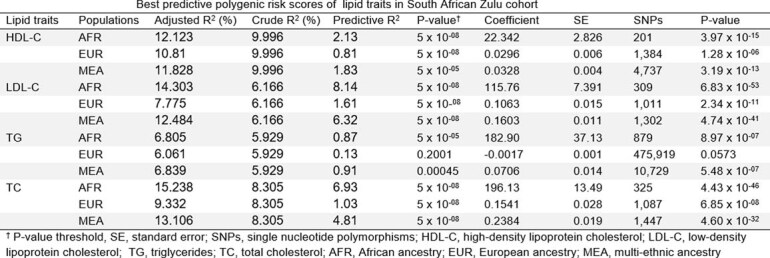
Best predictive polygenic risk scores of lipid traits in the South African Zulu cohort

**Extended Data Table 3:**
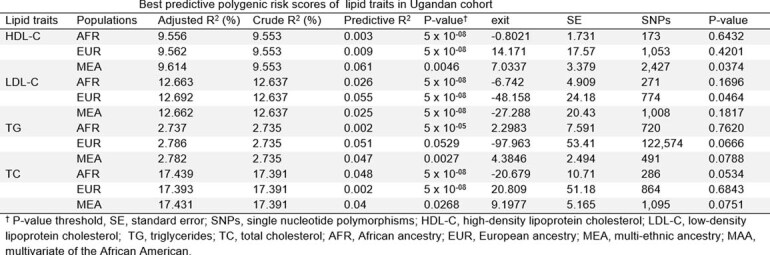
Best predictive polygenic risk scores of lipid traits in the Ugandan cohort

## Data Availability

Requests for resources and information should be directed to and will be fulfilled by the lead contact, S.F. (segun.fatumo@mrcuganda.org; segun.fatumo@lshtm.ac.uk). All individual-level data and phenotype, genotype and sequence data are available under managed access to researchers. Requests for access will be granted for all research consistent with the consent provided by participants. This would include any research in the context of health and disease that does not involve identifying the participants in any way. The array data have been deposited at the European Genome-phenome Archive (https://www.ebi.ac.uk/ega/, accession number EGAD00010000965). Requests for access to data may be directed to segun.fatumo@mrcuganda.org. Applications are reviewed by a data access committee, and access is granted if the request is consistent with the consent provided by participants. The data producers may be consulted by the data access committee to evaluate potential ethical conflicts. Requestors also sign an agreement that governs the terms on which access to data is granted. The genome-wide association summary statistics data are currently at https://www.ncbi.nlm.nih.gov/projects/gap/cgi-bin/study.cgi?study_id=phs001672.v3.p1#:~:text=MVP%20is%20an%20ongoing%20prospective,health%20and%20disease%20among%20veterans.dbGaP Study Accession: phs001672.v3.p1. The data used to construct the PRS are available on the PGS catalog: https://www.pgscatalog.org/publication/PGP000313/ (PGS ID accession: PGP000313).

## Code availability

We used publicly available software PRSice to compute PRS and its code is publicly available at https://www.prsice.info/. Other software programs used are listed and described in Methods.
