# Supplementary material for: Transferability of genetic risk scores in African populations
Source: Nat Med. 2022 Jun 2;28(6):1163–6. doi: 10.1038/s41591-022-01835-x (PMC9205766; doi:10.1038/s41591-022-01835-x)
Supplement: Supplementary file 1 — Reporting Summary [file 41591_2022_1835_MOESM1_ESM.pdf]

nature portfolio

Corresponding author(s): Segun Fatumo

Last updated by author(s): Mar 15, 2022

Reporting Summary

Nature Portfolio wishes to improve the reproducibility of the work that we publish. This form provides structure for consistency and transparency in reporting. For further information on Nature Portfolio policies, see our Editorial Policies and the Editorial Policy Checklist.

Statistics

For all statistical analyses, confirm that the following items are present in the figure legend, table legend, main text, or Methods section.

n/a	Confirmed	
☐	☒	The exact sample size (*n*) for each experimental group/condition, given as a discrete number and unit of measurement
☐	☒	A statement on whether measurements were taken from distinct samples or whether the same sample was measured repeatedly
☐	☒	The statistical test(s) used AND whether they are one- or two-sided<br>*Only common tests should be described solely by name; describe more complex techniques in the Methods section.*
☐	☒	A description of all covariates tested
☐	☒	A description of any assumptions or corrections, such as tests of normality and adjustment for multiple comparisons
☐	☒	A full description of the statistical parameters including central tendency (e.g. means) or other basic estimates (e.g. regression coefficient) AND variation (e.g. standard deviation) or associated estimates of uncertainty (e.g. confidence intervals)
☐	☒	For null hypothesis testing, the test statistic (e.g. *F*, *t*, *r*) with confidence intervals, effect sizes, degrees of freedom and *P* value noted<br>*Give P values as exact values whenever suitable.*
☒	☐	For Bayesian analysis, information on the choice of priors and Markov chain Monte Carlo settings
☒	☐	For hierarchical and complex designs, identification of the appropriate level for tests and full reporting of outcomes
☐	☒	Estimates of effect sizes (e.g. Cohen's *d*, Pearson's *r*), indicating how they were calculated

Our web collection on statistics for biologists contains articles on many of the points above.

Software and code

Policy information about availability of computer code

Data collection	No software was used to collect data.
Data analysis	We used publicly available software PRSice to compute PRS and its code is publicly available at https://www.prsice.info/. Other software programs used are listed and described in the Methods section of the manuscript.

For manuscripts utilizing custom algorithms or software that are central to the research but not yet described in published literature, software must be made available to editors and reviewers. We strongly encourage code deposition in a community repository (e.g. GitHub). See the Nature Portfolio guidelines for submitting code & software for further information.

Data

Policy information about availability of data

All manuscripts must include a data availability statement. This statement should provide the following information, where applicable:

- Accession codes, unique identifiers, or web links for publicly available datasets
- A description of any restrictions on data availability
- For clinical datasets or third party data, please ensure that the statement adheres to our policy

All genome-wide association summary statistics data are currently publicly available either at https://www.ebi.ac.uk/gwas/downloads/summary-statistics and/or https://dbgap.ncbi.nlm.nih.gov/. The multi-ethnic and African American PRS's will be made publicly available on the Polygenic Score (PGS) catalogue https://www.pgscatalog.org/. All individual-level data, phenotype, genotype and sequence data are available under managed access to researchers via the European Genome-phenome Archive (EGA) EGAD00010000965. Requests for access to the phenotypic data will be granted for all research consistent with the consent provided by participants. This would include any research in the context of health and disease that does not involve identifying the participants in any way.

Field-specific reporting

Please select the one below that is the best fit for your research. If you are not sure, read the appropriate sections before making your selection.

☒ Life sciences ☐ Behavioural & social sciences ☐ Ecological, evolutionary & environmental sciences

For a reference copy of the document with all sections, see nature.com/documents/nr-reporting-summary-flat.pdf

Life sciences study design

All studies must disclose on these points even when the disclosure is negative.

Sample size	Uganda genome resource (UGR) is the genomic, and phenotypic resource generated from the Uganda General Population Cohort (GPC). The GPC is a population-based cohort study founded in late 1980, and it has over 22,000 participants from 25 neighbouring villages in Kyamilibwa in rural Uganda. Of these individuals 6,407 consented for genetic study and were available and included in this study.
Data exclusions	No sample excluded from this study
Replication	We performed replication to assess the performance, portability and predictivity of African Americans derived GRS in 2,598 South African Zulu cohort
Randomization	Not applicable as this is not a therapeutic randomization study
Blinding	Not applicable as this is not a therapeutic randomization study

Reporting for specific materials, systems and methods

We require information from authors about some types of materials, experimental systems and methods used in many studies. Here, indicate whether each material, system or method listed is relevant to your study. If you are not sure if a list item applies to your research, read the appropriate section before selecting a response.

Materials & experimental systems

n/a	Involved in the study	
☒	☐	Antibodies
☒	☐	Eukaryotic cell lines
☒	☐	Palaeontology and archaeology
☒	☐	Animals and other organisms
☐	☒	Human research participants
☒	☐	Clinical data
☒	☐	Dual use research of concern

Methods

n/a	Involved in the study	
☒	☐	ChIP-seq
☒	☐	Flow cytometry
☒	☐	MRI-based neuroimaging

Human research participants

Policy information about studies involving human research participants

Population characteristics	Overall, our study had 6,407 Ugandan and 2,598 South African Zulu cohorts . The majority of our study participants were females; rural Ugandan (n=3,660; 57.1%) from the Uganda Genome Resource (UGR) cohort and South African Zulu (n=1,919; 73.8%) cohort. Our study participants mean ages were and 34.1 (18.3) years in the Ugandan cohort and 33.1 (15.1) years in the South African Zulu cohort. Mean serum lipid was 1.01 mmol/L, 2.05 mmol/L, 1.17 mmol/L and 3.57 mmol/L, for HDL-C, LDL-C. TG and TC, respectively in the UGR cohort and the corresponding values were respectively 1.08 mmol/L, 1.99 mmol/L, 1.60 mmol/L and 4.37 mmol/L in the South African Zulu study cohort.
Recruitment	From 2010-2011, the research questions have included the epidemiology and the genetics of communicable and non-communicable diseases (NCDs) to address the limited data on the burden and risk factors of NCDs in sub-Saharan Africa. The cohort comprises all residents (52% aged > 13 years, men and women in equal proportions) within one-half of a rural sub-county, residing in scattered houses, and largely farmers of three major ethnic groups.<br><br>Data collection was conducted from 2009 to 2013 for the DCC and from 2013 to 2014 for the DDS. The survey questinnaire included socioeconimic factors, health information, lifestyle factors, anthropometric measurements (including height, weight, systolic, diastolic blood pressure, and hip and waist circumference), biomarkers for communicable and non-communicable diseases, and genetic data.
Ethics oversight	The Uganda GPC was approved by Uganda Virus Research Institute Research and Ethics Committee (UVRI-REC #HS 1978) and the Uganda National Council for Science and Technology (UNCST #SS 4283).

The DDS was approved by the University of KwaZulu-Natal Biomedical Research Ethics Committee (UKZN BREC) (BF030/12) and the UK National Research Ethics Service (14/WM/); the DCC was approved by UKZN BREC (BF078/08) and the UK National Research Ethics Service (11/H0305/6).

Note that full information on the approval of the study protocol must also be provided in the manuscript.
